# Electroacupuncture repairs intestinal barrier by upregulating CB1 through gut microbiota in DSS-induced acute colitis

**DOI:** 10.1186/s13020-023-00733-9

**Published:** 2023-03-10

**Authors:** Jingze Yang, Lingli Wang, Minhui Mei, Jinlu Guo, Xin Yang, Shi Liu

**Affiliations:** Department of Gastroenterology, Union Hospital, Tongji Medical College, Huazhong University of Science and Technology, Wuhan, 430022 China

**Keywords:** Electroacupuncture, Intestinal barrier, CB1, Gut microbiota, Autophagy

## Abstract

**Background:**

A few studies have reported that electroacupuncture (EA) can repair the intestinal barrier through unknown mechanisms. Cannabinoid receptor 1 (CB1) was shown to play an important role in the protection of the gut barrier in recent studies. Gut microbiota can influence the expression of CB1. In this study, we explored the effect of EA on the gut barrier in acute colitis and its mechanism.

**Methods:**

A dextran sulfate sodium (DSS)-induced acute colitis model, CB1 antagonist model and fecal microbiota transplantation (FMT) model were used in this study. The disease activity index (DAI) score, colon length, histological score, and inflammatory factors were detected to evaluate colonic inflammation. Methods for detecting intestinal barrier functions included the expression of tight junction proteins, intestinal permeability, and the number of goblet cells. Moreover, 16S rRNA sequencing was applied to analyze alterations in the gut microbiota. Western blotting and RT-PCR were performed to assess the levels of CB1 and autophagy-related proteins. Autophagosomes were observed by transmission electron microscopy.

**Results:**

EA reduced the DAI score, histological score, levels of inflammatory factors, and restored the colon length. Moreover, EA increased the expression of tight junction proteins and the number of goblet cells, and decreased intestinal permeability. In addition, EA remodeled the community structure of the gut microbiota, increased the expression of CB1, and enhanced the degree of autophagy. However, the therapeutic effects were reversed by CB1 antagonists. In addition, FMT in the EA group exhibited similar effects to EA and upregulated CB1.

**Conclusions:**

We concluded that EA may protect intestinal barrier functions by increasing the expression of CB1 to enhance autophagy through gut microbiota in DSS-induced acute colitis.

**Supplementary Information:**

The online version contains supplementary material available at 10.1186/s13020-023-00733-9.

## Background

The intestinal barrier is the first line of defense to protect the intestine from the invasion of bacteria or noxious antigens. Intestinal barrier disruption is an important cause of the occurrence and development of ulcerative colitis [1]. However, effective interventions to repair the gut barrier are lacking. Electroacupuncture (EA), which combines electrical stimulation and acupuncture, has been reported to restore the gut barrier in multiple animal models of inflammation with safety and good reproducibility [2, 3]. Recently, some studies found that EA could protect the intestinal barrier and alleviate intestinal inflammation in dextran sulfate sodium (DSS)-induced colitis [4, 5], but the mechanisms involved remained unclear.

Cannabinoid receptor 1 (CB1) is an important component of the endocannabinoid system, which has been proven to restore the blood–brain barrier and relieve neuroinflammation [6, 7]. In recent years, the effect of CB1 on repairing the intestinal barrier has gradually been discovered. A review demonstrated that activation of CB1 could repair the intestinal barrier and decrease intestinal permeability, thereby relieving intestinal inflammation [8]. Autophagy is a biological process in which eukaryotic cells use lysosomes to degrade organelles and proteins and is closely related to the maintenance of gut barrier functions [9, 10]. CB1 was reported to participate in the regulation of the expression of autophagy-related proteins and further influence the degree of autophagy [11]. The effects of EA on CB1 have been verified in several animal models [12, 13], but no study has investigated the role of EA on CB1 in colitis. Thus, whether EA can protect the intestinal barrier by regulating the level of CB1 to enhance autophagy needs to be further investigated.

How EA regulates the level of CB1 should be investigated. Some researchers have noted the correlation between gut microbiota and CB1. Antibiotic treatment decreased the level of CB1 compared with the control [14]. Accordingly, probiotic supplementation upregulated the expression of CB1 [15]. Notably, the effects of EA on gut microbiota have been proven [16, 17], and our previous study also demonstrated that EA could restore the gut microbiota in DSS-induced colitis [5]. Therefore, we hypothesized that EA could increase the level of CB1 by regulating the gut microbiota.

In this study, we aimed to evaluate whether EA can upregulate CB1 to enhance the degree of autophagy, thereby repair the intestinal barrier and ultimately improve intestinal inflammation in DSS-induced acute colitis.

## Methods

### Experimental animals

Eight-week-old male C57Bl/6J mice were obtained from Wei Tong Li Hua Biotechnology Co. (Beijing, China) and housed in a specific-pathogen-free facility (22 °C, 12 h/12 h light–dark cycle) in the laboratory animal center of Tongji Medical College. All mice were allowed to adapt to the laboratory conditions for 2 weeks with free access to food and sterile water. The animal studies were approved by the Animal Ethics Committee of Tongji Medical College of Huazhong University of Science and Technology, and the works were conducted in accordance with the principles of the Animal Care and Use Committee of this institution.

### Experimental protocols

**Fig. 1:**
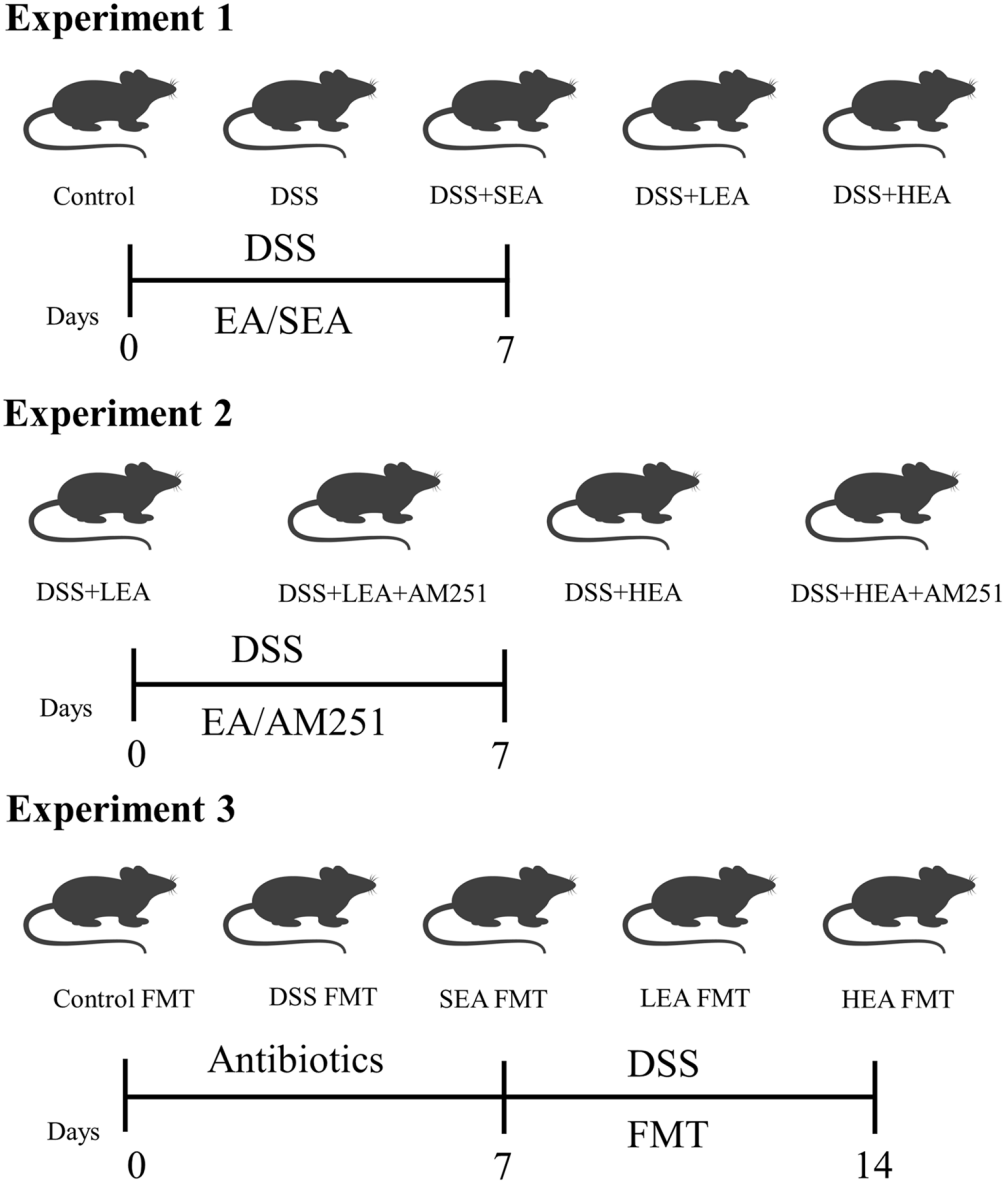
The grouping and experimental procedure. Experiment 1. DSS-induced acute colitis model and EA treatment process. Experiment 2. DSS-induced acute colitis model, EA treatment process and AM251-treated. Experiment 3. Antibiotics-treated colitis model and FMT process

This study had three parts. The grouping and experimental procedure are shown in Fig. 1.

1. A DSS-induced acute colitis model and EA model were established to explore whether EA could repair the gut barrier in acute colitis. Mice were randomly distributed into 5 groups: the control group, DSS-induced colitis group (DSS), DSS-induced colitis treated with sham EA group (DSS + SEA, only acupuncture without an electric current), DSS-induced colitis treated with low-frequency EA group (DSS + LEA, 10 Hz, 1 mA), and DSS-induced colitis treated with high-frequency EA group (DSS + HEA, 100 Hz, 1 mA). All mice except the controls received 3% (w/v) DSS (MP Biomedical, 36,000–50,000 MW, Solon OH, USA) in their drinking water for 7 days. The mice in the control group were given normal water. Moreover, EA treatments were administered from Day 1 to Day 7.
2. A CB1 antagonist (AM251) was used to investigate the role of CB1 in the therapeutic effects of EA. The mice were randomly divided into four groups: the DSS + LEA group, DSS + LEA + AM251 group, DSS + HEA group, and DSS + HEA + AM251 group. AM251 was dissolved in DMSO and then diluted with PEG 300, Tween 80 and saline. The mice from the AM251 group received the CB1 antagonist (5 mg/kg) by intraperitoneal injection daily for 7 days, while the control group received equal doses of solvents. Similarly, all mice were given 3% DSS to induce acute colitis and EA was conducted 30 min after intraperitoneal injection.
3. Fecal microbiota transplantation (FMT) was performed to explore the role of gut microbiota in the beneficial effects of EA. The mice were treated with a cocktail of antibiotics by oral gavage (200 µl per mouse) every 12 h for 7 days to prepare germ-free mice and then used as recipients. Antibiotics included vancomycin (100 mg/kg), ampicillin (200 mg/kg), neomycin (200 mg/kg), and metronidazole (200 mg/kg). The mice from the first part were used as donors and their feces were collected to prepare fecal suspensions (one stool pellet was dissolved in 2 ml of sterile phosphate buffer saline). Mice were randomly distributed into 5 groups: the control FMT group (fecal suspensions from the control group), DSS FMT group (fecal suspensions from the DSS group), SEA FMT group (fecal suspensions from the DSS + SEA group), LEA FMT group (fecal suspensions from the DSS + LEA group), and HEA FMT group (fecal suspensions from the DSS + HEA group). Then, 3% DSS was used to induce acute colitis and the fecal suspensions were transplanted into germ-free mice by oral gavage (0.2 ml per mouse) once a day for 7 days.

The mice were weighed, and stool consistency and rectal bleeding were observed daily to calculate the disease activity index (DAI) scores as previously described [18]. Mice were sacrificed on Day 8, and colon length was measured. The colon tissues from some mice were used immediately to assess the intestinal permeability (n = 6/group) and the others (n = 6/group) were fixed, embedded and stored at − 80 °C.

### EA

The bilateral ST36 acupoints, located on the posterolateral aspect of the knee approximately 2 mm below the fibular head, were selected for EA in our study. The mice were acupunctured by a pair of stainless needles in ST36 acupoints at a depth of 2–3 mm on bilateral hind limbs. An electrical stimulator (G6805-2A; Shanghai Huayi Medical Instrument Co., Ltd., Shanghai, China) was used in the experiment and stimulation was sustained for 30 min every day. For the SEA group, the mice underwent the same process without an electrical current. Mice were placed in a stimulus fixator for 30 min/day for 1 week before the experiment to eliminate constraint pressure.

### Histological examination

The distal colon specimens were fixed with 4% paraformaldehyde for 24 h and embedded in paraffin. Then, paraffin-embedded colonic tissues were sectioned (4 μm in thickness), stained with hematoxylin and eosin, and analyzed by a pathologist without information on the experimental procedures based on the scoring criteria as described previously [19].

### Goblet cell number counting

The colonic segments were fixed in Methanol-Carnoy solution (methanol: chloroform: glacial acetic acid = 6:3:1), paraffin embedded and cut into serial 4 μm sections. Then, goblet cell staining was performed via Alcian blue/periodic acid-Schiff staining according to the instructions. The pathologist, without knowledge of any experimental procedures, counted the number of goblet cells.

### Intestinal permeability assessment

Intestinal permeability was determined using Ussing chamber analysis. The colonic mucosa was intactly scraped from the distal colon specimens, installed in a slider with a 0.3 cm^2^ rectangular hole in the center, fixed in the U-shaped chamber, and immersed in oxygen-containing Krebs’ solution on both the serosal and mucosal sides. Then, the chamber was mounted on Ussing Chamber System (World Precision Instruments, USA). The transepithelial resistance (TER) of the colonic mucosa was recorded by an automatic voltage clamp model after a 20 min equilibration. In addition, mucosal-to-serosal permeability was assessed by fluorescein isothiocyanate conjugated dextran (FD4, FITC-dextran, molecular weight: 4 kD, Sigma-Aldrich, Madrid, Spain). After the TER recording, 1 mg/ml FD4 was added to the mucosal side of the chamber, and the same volume of Krebs’ solution was added to the serosal side without light. One hundred microliters of solution was sampled from the serosal side every 30 min over a 2 h period, and the fluorescence intensity was detected by a fluorescence spectrophotometer (485 nm/528 nm, Ex/Em, BioTek, Winooski, VT, USA). The FD4 concentration in the serosal side was evaluated by a standard curve of continuous dilutions of FD4 in Krebs’ solution.

### Transmission electron microscopy

Colonic tissues were fixed with 2.5% glutaraldehyde. After removal of excess fixative with PBS, samples were fixed with 1% osmic acid at 20 °C for 2 h, dehydrated in acetone, and then infiltrated with acetone and epoxy. Epoxy resin-embedded tissues were sectioned (80 nm in thickness), stained with uranyl acetate and lead citrate, and finally viewed under a transmission electron microscope (FEI, Hillsboro, America).

### 16S rRNA sequencing

Feces were collected from mice in Part 1 at Day 8 for microbiota assessment. Fecal microbial DNA was extracted from the above feces and then subjected to 16S rRNA sequencing on an Illumina MiSeq platform (Illumina, USA) as previously described [20]. The microbial composition and biodiversity were assessed. In addition, the relative abundances of specific families and genera were further analyzed. To assess the effect size of each differentially abundant taxon, Linear discriminant analysis (LDA) effect size was performed by non-parametric factorial Kruskal–Wallis (KW) sum-rank test. Moreover, PICRUSt software was applied to obtain functional predictions, and the taxonomic classification of sequences was conducted based on the Kyoto Encyclopedia of Genes and Genome (KEGG). Finally, correlation analysis was carried out to show the relationship between the expression of CB1 and microbial abundance.

### Western blot analysis

Proteins were extracted from colon tissues with RIPA lysis buffer (Beyotime, Shanghai, China) containing phenylmethyl sulfonyl fluoride. A bicinchoninic acid protein assay kit (Beyotime) was used to measure the concentration of protein. Suitable quality protein samples were subjected to sodium dodecyl sulfate-polyacrylamide gel electrophoresis and transferred to PVDF membranes (Millipore, Billerica, MA, USA). The PVDF membranes were blocked with 8% skim milk at room temperature for 1 h and incubated with primary antibodies at 4 °C overnight. The corresponding secondary antibodies were used at room temperature for 1 h, and the protein signals were detected with a FluorChem FC3 system (ProteinSimple, California, USA) using an enhanced chemilusystem reagent (Thermo Fisher, Waltham, USA) according to the manufacturer’s instructions. The antibodies used in this study were as follows: TNFα (1:200, Santa Cruz Biotechnology, Texas, USA), IL-1β (1:1000, Cell Signaling Technology, Massachusetts, USA), IL6 (1:200, Santa Cruz Biotechnology), ZO-1 (1:1000, Invitrogen, California, USA), Occludin (1:1000, Invitrogen), P62 (1:10,000, Abcam, Cambridge, USA), LC3 (1:1000, Proteintech, Chicago, America), CB1 (1:1000, Proteintech), GAPDH (Abclonal, Wuhan, China), horseradish peroxidase (HRP)-linked goat anti-rabbit IgG, and HRP-linked goat anti-mouse IgG (1:4000, Antgene, Wuhan, China).

### RNA extraction and quantitative real-time PCR (RT-PCR) analysis

Total RNA was isolated from colonic tissues by TRIzol reagent (TaKaRa, Otsu, Japan) according to the manufacturer’s protocol. Then, cDNA synthesis was carried out by using the PrimeScript™ RT Master Mix Kit (TaKaRa). RT-PCR was performed using SYBR-Green PCR master mix (TaKaRa) in the Roche Light Cycler R480 system (Roche, Basel, Switzerland). The relative expression of mRNA level was calculated by 2^−ΔΔCT^. The primers used in this study are shown in Table 1.

**Table 1:** Primer sequences used for RT-PCR in this study

Gene	Forward (5′–3′)	Reverse (5′–3′)
GAPDH	AGGTCGGTGTGAACGGATTTG	TGTAGACCATGTAGTTGAGGTCA
IL6	CCGGAGAGGAGACTTCACAG	CAGAATTGCCATTGCACAAC
TNFα	CTTGGAAATAGCTCCCAGAA	CATTTGGGAACTTCTCATCC
IL1β	GGAGAGCCCTGGATACCAAC	CAGGGTCCCAGACAGAAGTT
ZO-1	GCTTTAGCGAACAGAAGGAGC	TTCATTTTTCCGAGACTTCACCA
Occludin	TGAAAGTCCACCTCCTTACAGA	CCGGATAAAAAGAGTACGCTGG
CB1	GTACCATCACCACAGACCTCCTC	GGATTCAGAATCATGAAGCATCCA

*RT-PCR* real-time PCR

### Immunofluorescence

Paraffin-embedded sections were dewaxed, hydrated, treated for antigen retrieval, and blocked with 10% donkey serum. Then, the primary antibodies were used for the incubation of the sections at 4 °C overnight. The next day, the slides were stained with relevant secondary antibodies at room temperature for 1 h. Nuclei were stained with DAPI (Servicebio, Wuhan, China). Finally, the sections were observed with a confocal microscope (Olympus, Tokyo, Japan) after sealing with an anti-fluorescence quencher. The antibodies used in this study were as follows: anti-ZO-1 (1:200, Genetex, Texas, America), anti-Occludin (1:200, Genetex), and Alexa Fluor 488-conjugated donkey anti-rabbit IgG (1:200, Antgene, Wuhan, China).

### Statistical analysis

Statistical analysis was carried out using SPSS 22 software and figures were designed by GraphPad software. Data are expressed as the means ± SEMs. For comparisons between two groups, a two-tailed unpaired Student’s t test was used, and one-way analysis of variance (ANOVA) was performed for multiple group comparisons. P values < 0.05 were considered statistically significant.

## Results

### EA alleviated colonic inflammation

**Fig. 2:**
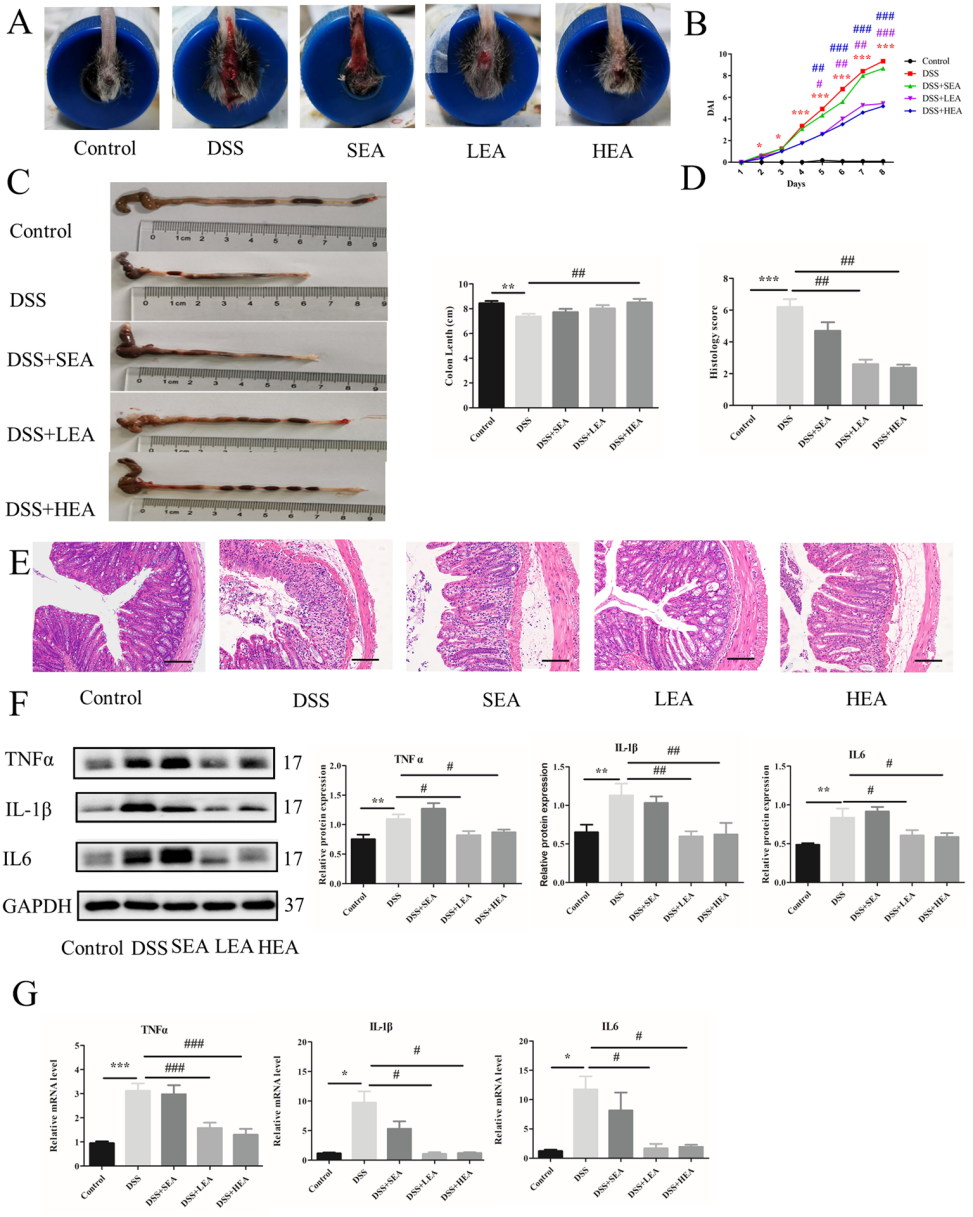
EA alleviated intestinal inflammation in DSS-induced acute colitis. **A** A representation of rectal bleeding in mice at Day 8. **B** Disease activity index scores from Day1 to Day 8. **C** Colon length of mice. **D** Histological scores. **E** Representative HE staining images. Scale bar = 100 μm. **F** Colonic protein levels of TNFα, IL1β and IL6. **G** Relative mRNA expression of colonic TNFα, IL1β and IL6. Data are expressed as the mean ± SEM (n = 12 mice per group in **B**, **C**, n = 6 mice per group in **D**, **F**, **G** *P < 0.05, **P < 0.01, ***P < 0.001 vs. the control group; ^#^P < 0.05, ^##^P < 0.01, ^###^P < 0.001 vs. the DSS group)

Rectal bleeding in the DSS group was more serious than that in the control group, while the mice in the EA group showed obvious improvement (Fig. 2A). Compared with those of the control group, the DAI scores of the DSS group were increased from Day 2. The DAI scores of the EA group were decreased from Day 5 compared to those of the DSS group (Fig. 2B). For colon length (Fig. 2C), the DSS group had a shorter colon (P = 0.003) than the control group, and the HEA group had a longer colon (P = 0.002) than the DSS group, while the LEA group had an increasing trend (P = 0.068). In addition, DSS exposure induced severe colon tissue damage, such as the disappearance of crypt structure, intestinal epithelium destruction, submucosal edema, inflammatory cell infiltration, and significantly increased histological scores (P < 0.001) compared with the control, and EA treatment improved the injuries, and reduced these scores (P = 0.002 in the LEA group and P = 0.002 in the HEA group, Fig. 2D, E). Western blotting analysis showed that the expression levels of TNF α, IL-1β, and IL6 were increased in the DSS group (P = 0.004, P = 0.007, and P = 0.002, respectively), but decreased in the LEA (P = 0.016, P = 0.003, and P = 0.029, respectively) and HEA groups (P = 0.046, P = 0.005, and P = 0.02, respectively; Fig. 2F). In addition, the mRNA levels of TNF α, IL-1β, and IL6 were increased with DSS exposure (P < 0.001, P = 0.04, and P = 0.034, respectively), and compared with the mice in the DSS group, the mice in the LEA (P < 0.001, P = 0.038, and P = 0.036, respectively) and HEA groups (P < 0.001, P = 0.042, and P = 0.045, respectively) had reduced levels of TNF α, IL-1β, and IL6 (Fig. 2G).

### EA repaired the intestinal barrier

**Fig. 3:**
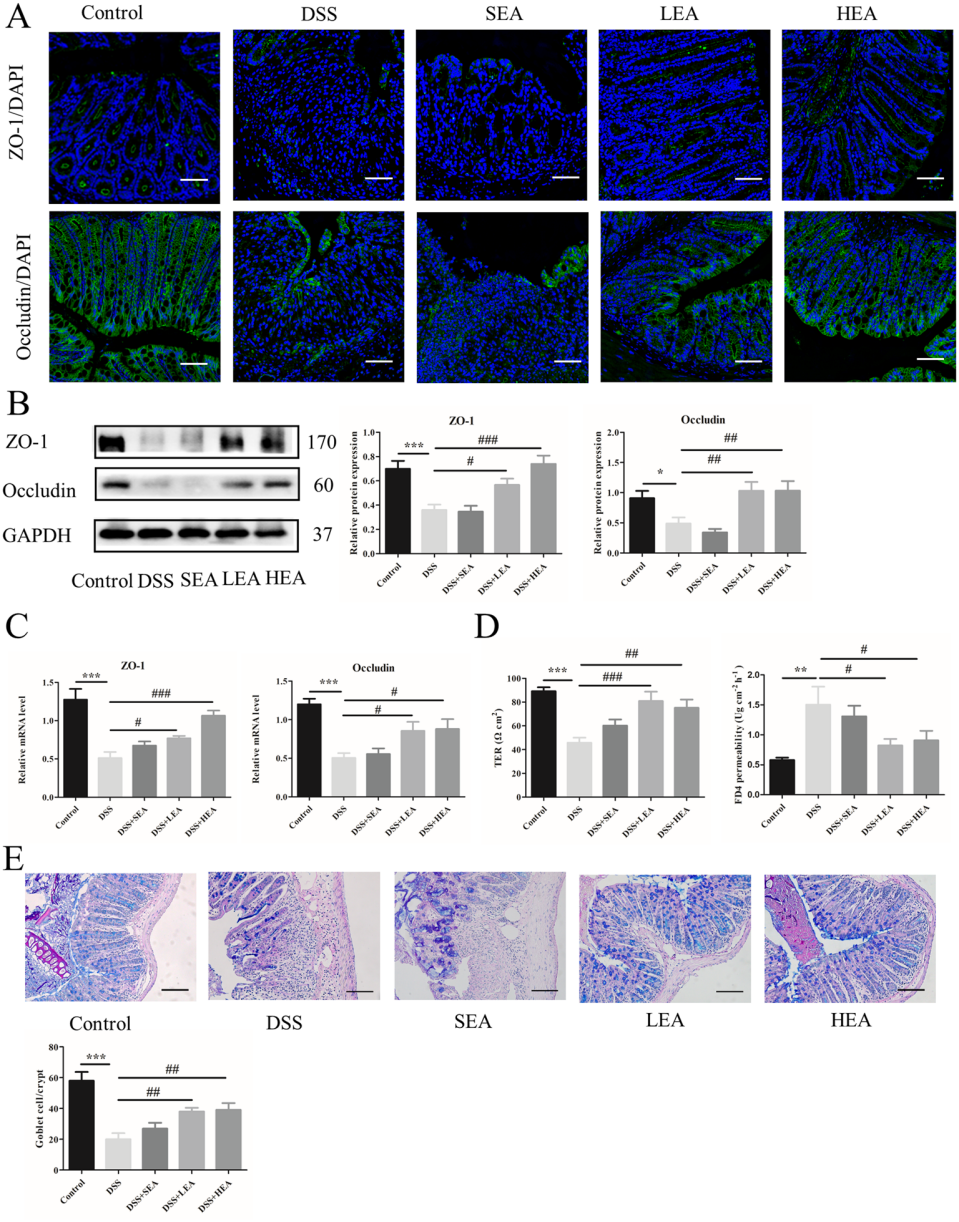
EA improved the integrity of the intestinal barrier. **A** The distribution of ZO-1, and Occludin in colonic tissues. Scale bar = 100 μm. **B** The protein levels of colonic ZO-1 and Occludin. **C** The mRNA expression of colonic ZO-1 and Occludin. **D** Transepithelial resistance and FD4 permeability in the colon. **E** The number of goblet cells in the colon. (N = 6 mice per group, *P < 0.05, **P < 0.01, ***P < 0.001 vs. control group; ^#^P < 0.05, ^##^P < 0.01, ^###^P < 0.001 vs. the DSS group)

Immunofluorescence (IF) staining demonstrated that the distribution of ZO-1 and Occludin was lessened in the DSS group. Both LEA and HEA promoted their expression (Fig. 3A). The protein levels of ZO-1 and Occludin were also reduced in the mice of the DSS group (P < 0.001 and P = 0.023 respectively), and EA increased the expression (P = 0.016 and P = 0.004 in the LEA group; P < 0.001 and P = 0.004 in the HEA group, respectively, Fig. 3B). Figure 3C showed that DSS treatment reduced the mRNA levels of ZO-1 and Occludin (P < 0.001 and P < 0.001 respectively), while EA partly restored the expression of ZO-1 and Occludin (P = 0.036 and P = 0.015 in the LEA group; P < 0.001 and P = 0.01 in the HEA group, respectively). The Ussing chamber analysis indicated that the TER of the mice was decreased with DSS (P < 0.001) and increased with DSS + EA (P < 0.001 in the LEA group and P = 0.001 in the HEA group). Moreover, EA treatment decreased FD4 permeability (P = 0.012 in the LEA group and P = 0.027 in the HEA group) compared with that in the DSS group (Fig. 3D). We also found that the number of goblet cells was decreased in the DSS-treated mice (P < 0.001), and EA treatment resulted in a partial recovery (P = 0.006 in the LEA group and P = 0.004 in the HEA group; Fig. 3E).

### EA elevated the expression of CB1

**Fig. 4:**
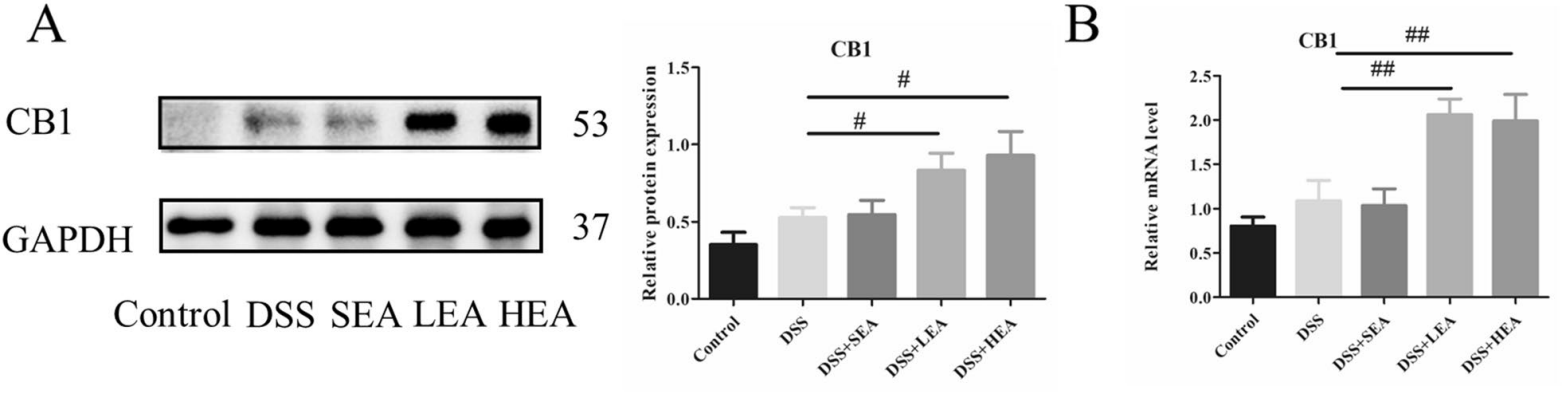
EA upregulated the expression of CB1. **A** The protein level of colonic CB1. **B** The mRNA expression of colonic CB1. (n = 6 mice per group, ^#^P < 0.05, ^##^P < 0.01 vs. the DSS group)

In Fig. 4, we detected the expression of CB1 and found no significant difference between the control and DSS groups at either the protein or mRNA level. However, the relative level of CB1 was increased in the LEA (P = 0.049) and HEA groups (P = 0.012) at the protein level compared to that of the DSS group. Similarly, the results of RT‒PCR revealed that EA-treatment upregulated the expression of CB1 (P = 0.003 in the LEA group and P = 0.005 in the HEA group).

### EA enhanced autophagy

**Fig. 5:**
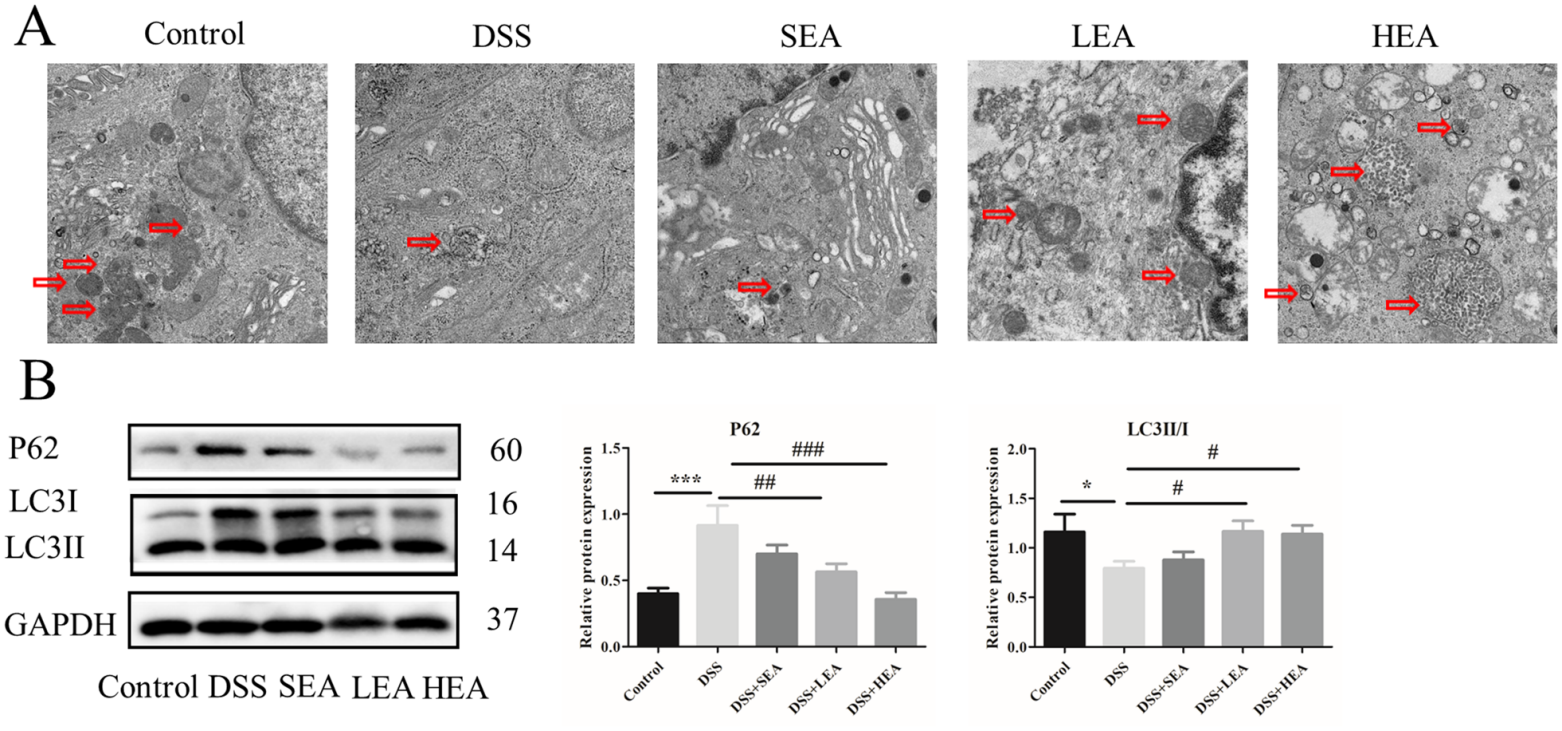
EA enhanced the degree of autophagy. **A** The autophagosome in the colon. **B** The protein levels of autophagy-related proteins in the colon. (n = 6 mice per group, *P < 0.05, ***P < 0.001 vs. the control group; ^#^P < 0.05, ^##^P < 0.01, ^###^P < 0.001 vs. the DSS group)

The TEM results revealed that the number of autophagosomes in the DSS group was decreased compared with that in the control group, and EA treatment increased the number of autophagosomes compared with that in the DSS group (Fig. 5A). For the autophagy related proteins, the relative expression of P62 was upregulated after oral administration of DSS (P < 0.001), and it was downregulated with EA treatment (P = 0.006 in the LEA group, and P < 0.001 in the HEA group). Moreover, the relative level of LC3II/I was reduced in the mice of the DSS group (P = 0.031), and it was increased in the EA group (P = 0.028 in the LEA group, and P = 0.041 in the HEA group; Fig. 5B).

### EA improved gut microbiota dysbiosis

**Fig. 6:**
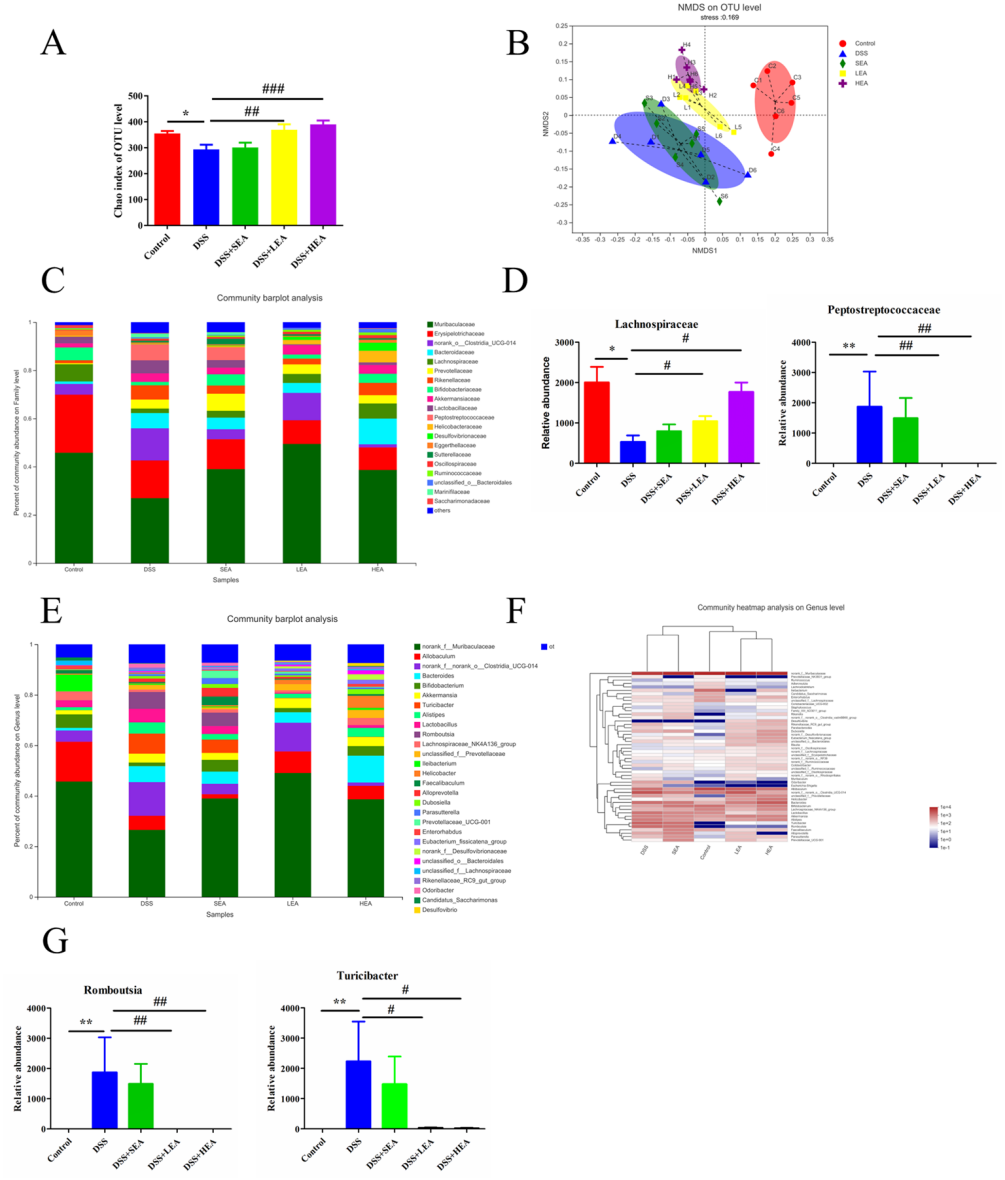
EA remodeled the gut microbiota. **A** The alpha diversity (Chao index). **B** The NMDS plots of unweighted UniFrac distances of beta diversity. **C**, **D** Relative abundance of microbes at the family level. **E**–**G** Microbial relative abundance at the genus level. (N = 6 mice per group, *P < 0.05, **P < 0.01 vs. the control group; ^##^P < 0.01, ^###^P < 0.001 vs. the DSS group)

**Fig. 7:**
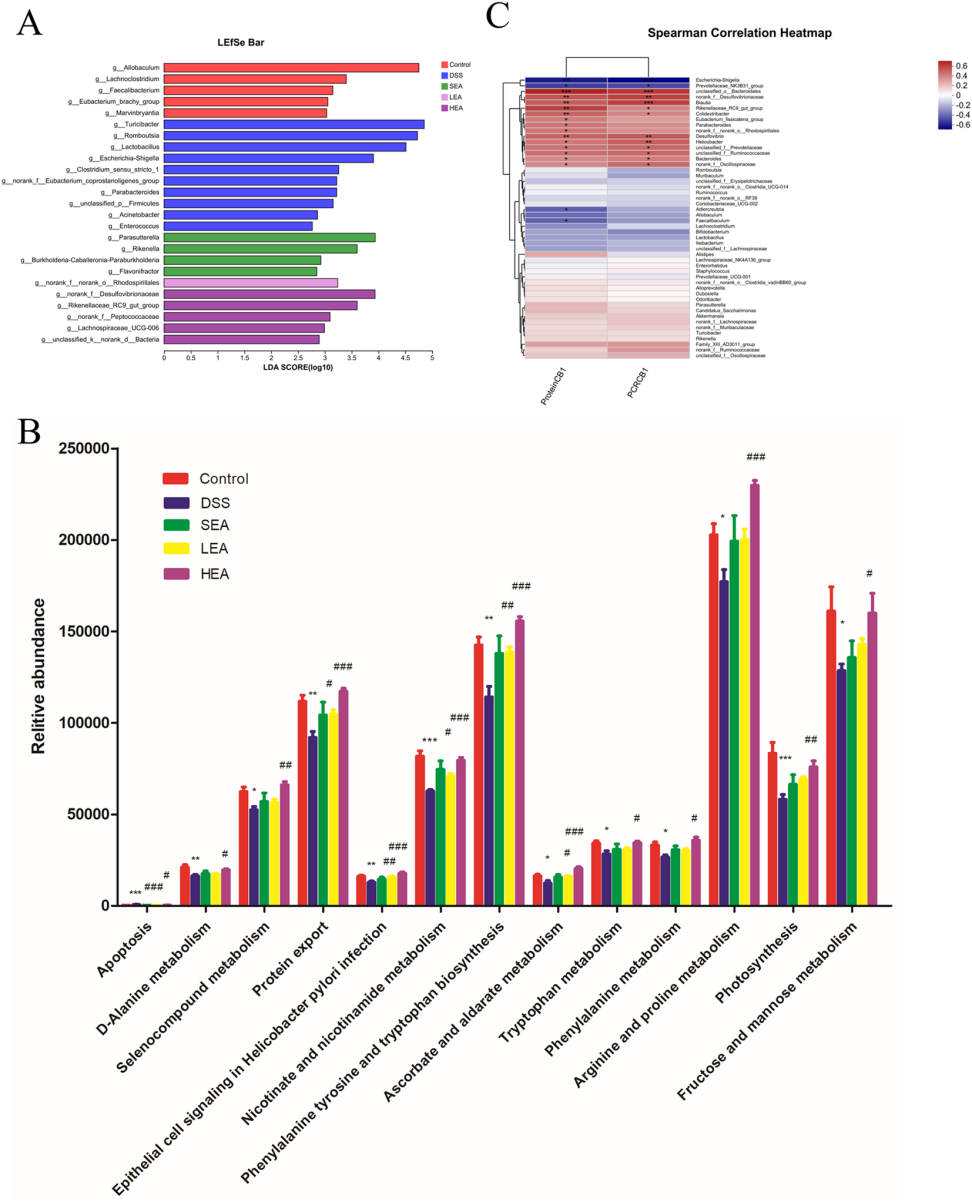
EA altered the composition and function of the gut microbiota. **A** LDA value distribution diagram among the groups. **B** KEGG metabolic pathway analysis among the groups. **C** The correlation heatmap of genera and the expression of CB1

Mice in the DSS group showed a significant decrease in alpha-diversity (Chao index; P = 0.015), and EA exposure resulted in an increase in the Chao index (P = 0.004 in the LEA group, P < 0.001 in the HEA group) compared to that of the DSS group (Fig. 6A). Unweighted UniFrac-based nonmetric multidimensional scaling demonstrated a significant separation between the DSS and control groups, whereas the distance between the EA and control groups was less than that between the DSS and control groups (Fig. 6B). At the family level (Fig. 6C, D), the relative abundance of *Lachnospiraceae* was decreased in the DSS group (P = 0.015) compared with the control group, while it was increased in the LEA (P = 0.029) and HEA group (P = 0.01) compared to the DSS group. However, the relative abundance of *Peptostreptococcaceae* was increased in the mice treated with DSS (P = 0.002) and decreased in the mice treated with LEA (P = 0.009) and HEA (P = 0.004). At the genus level (Fig. 6E–G), we found that microbiota in the EA group were more similar to those in the control group. In addition, the relative abundances of *Romboutsia* and *Turicibacter* were increased in the DSS group (P = 0.002; P = 0.002) compared with the control group, while they were decreased in the LEA (P = 0.009; P = 0.041) and HEA group (P = 0.004; P = 0.026) in comparison to the DSS group. LDA was then conducted to determine the taxa that most probably explain the differences among these groups (Fig. 7A). We detected the enrichment of norank_f_norank_o_*Rhodospirillales* in the LEA group; the profusion of norank_f_*Desulfovibrionaceae*, *Rikenellaceae*_RC9_gut_group, norank_f_*Peptococcaceae*, *Lachnospiraceae*_UCG-006, and unclassified_k_norank_d_*Bacteria* in the HEA group.

Subsequently, to assess the effects of EA on gut microbiota function, we performed KEGG metabolic pathway analysis. As shown in Fig. 7B, compared with the DSS group, the LEA and HEA group were significantly enriched with those microbial communities expressing functional genes associated with Protein export; Epithelial cell signaling in Helicobacter pylori infection; Nicotinate and nicotinamide metabolism; Phenylalanine, tyrosine and tryptophan biosynthesis; and Ascorbate and aldarate metabolism.

To determine whether alterations in the gut microbiota affected the expression of CB1, we performed Spearman correlation analysis to assess association between key genera and the levels of CB1. As shown in Fig. 7C, norank_f_*Desulfovibrionaceae*, norank_f_norank_o_*Rhodospirillales*, and *Rikenellaceae*_RC9_gut_group were strongly positively correlated with the expression of CB1 at both the protein and mRNA levels. Interestingly, LDA revealed that these genera were significantly enriched in the EA group, which indicates that the high enrichment of certain gut microbiota in the EA group may be associated with a high expression of CB1.

### AM251 reversed the therapeutic effects of EA on intestinal inflammation, the gut barrier, and autophagy

**Fig. 8:**
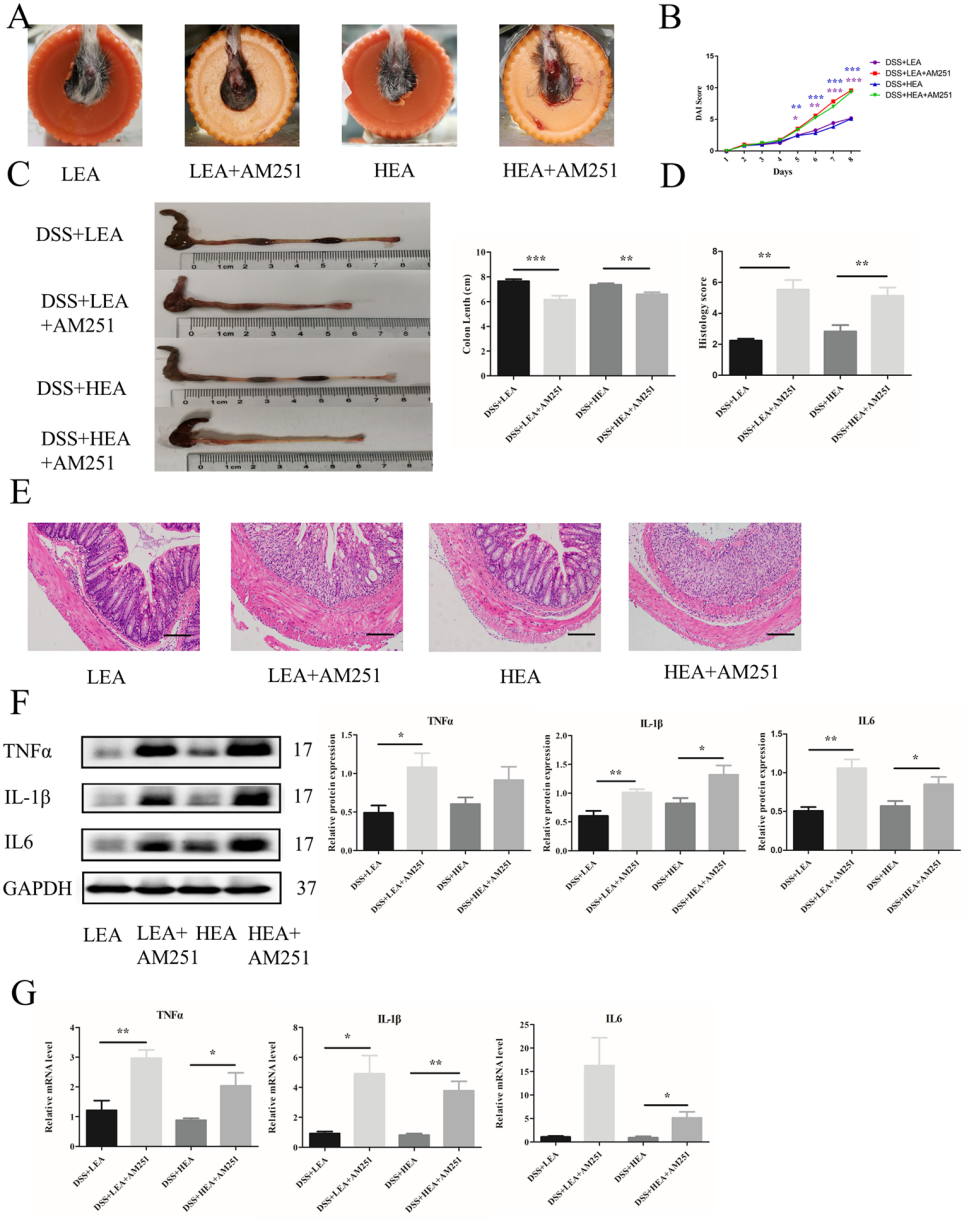
AM251 reversed the anti-inflammatory effects of EA. **A** The representation of rectal bleeding in mice at Day 8. **B** Disease activity index scores from Day 1 to Day 8. **C** Colon length of mice. **D** Histological scores. **E** Representative HE staining images. Scale bar = 100 μm. **F** Colonic protein levels of TNFα, IL1β and IL6. **G** Relative mRNA expression of colonic TNFα, IL1β and IL6. Data are expressed as the mean ± SEM (n = 12 mice per group in **B**, **C**, n = 6 mice per group in **D**, **F**, **G** *P < 0.05, **P < 0.01, ***P < 0.001 vs. the EA group)

To explore the role of CB1 in EA, we used AM251 (a CB1 selective antagonist) in this study, and the expression of CB1 showed a significant decrease at both the protein (P = 0.019 in the LEA + AM251 group and P = 0.001 in the HEA + AM251 group) and mRNA levels (P < 0.001 in the LEA + AM251 group and P = 0.001 in the HEA + AM251 group; Additional file 1: Fig. S1). As shown in Fig. 8A, rectal bleeding in the EA + AM251 group was more serious than that in the EA group. Similarly, the DAI scores in the EA + AM251 group were also increased compared to those in the EA group on Day 5 (Fig. 8B). The colon length in the mice treated with EA + AM251 was shorter (P < 0.001 in the LEA + AM251 group and P = 0.001 in the HEA + AM251 group, Fig. 8C). In addition, colon tissue damage (the destroyed intestinal epithelium, necrotic intestinal glands, and lots of infiltrated inflammatory cells) was severe, and the histological scores were increased (P = 0.002 in the LEA + AM251 group and P = 0.006 in the HEA + AM251 group) in the presence of AM251 (Fig. 8D, E). Regarding the expression of inflammatory factors, the protein level of TNFα was increased significantly in the LEA + AM251 group (P = 0.017) and showed an increasing trend in the HEA + AM251 group. Moreover, the protein levels of IL-1β and IL6 were increased (P = 0.003 and P = 0.001 in the LEA + AM251 group; P = 0.025 and P = 0.035 in the HEA + AM251 group, respectively) in the EA + AM251 group (Fig. 8F). Furthermore, the mRNA levels of TNFα and IL-1β were increased when the CB1 selective antagonist was administered (P = 0.002 and P = 0.022 in the LEA + AM251 group; P = 0.045 and P = 0.005 in the HEA + AM251 group, respectively). The mRNA level of IL6 was significantly increased in the mice in the HEA + AM251 group (P = 0.021) and exhibited an increasing trend in the LEA + AM251 group (Fig. 8G).

**Fig. 9:**
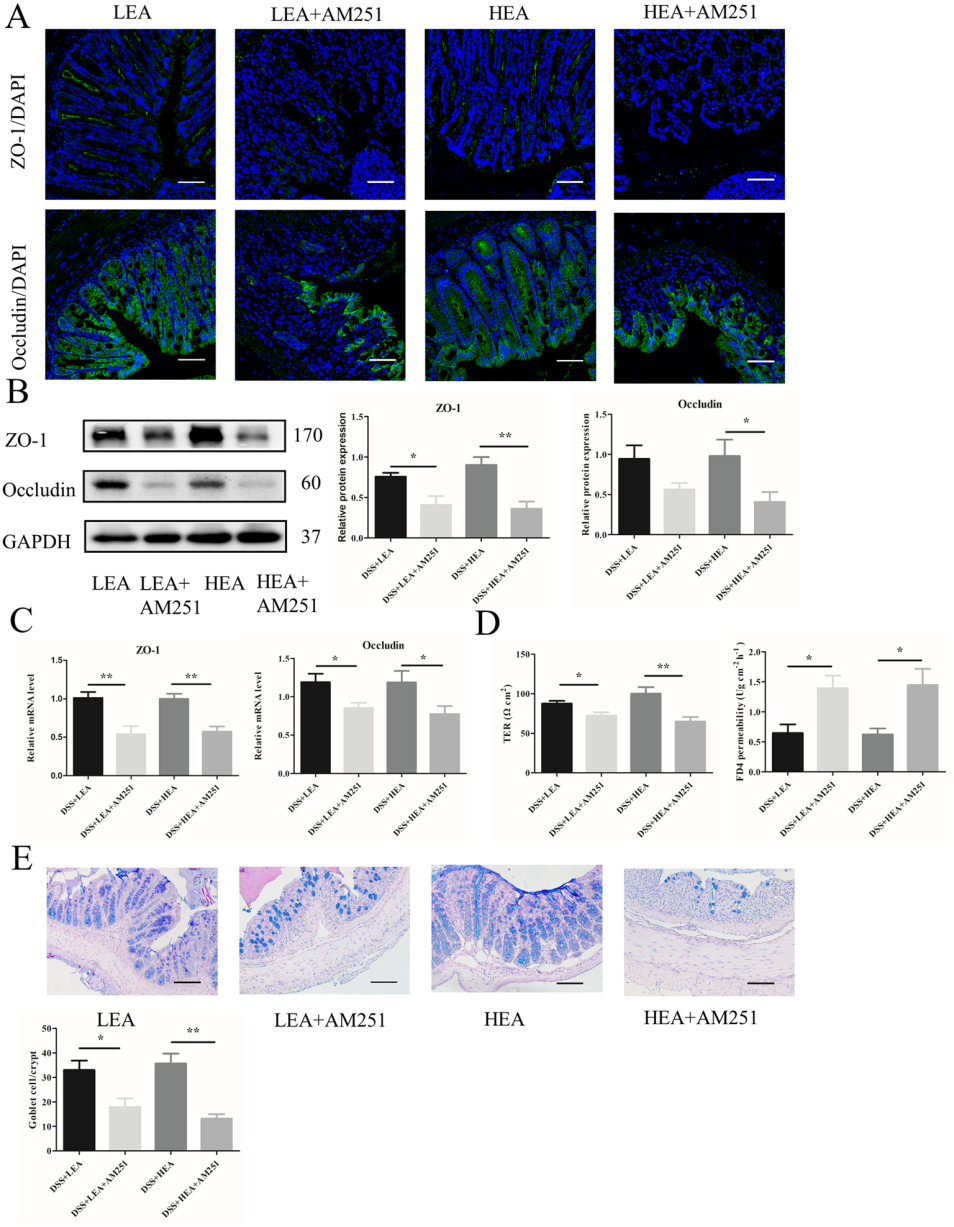
AM251 reversed the barrier-repairing effects of EA. **A** The distribution of ZO-1, and Occludin in colonic tissues. Scale bar = 100 μm. **B** The protein levels of colonic ZO-1 and Occludin. **C** The mRNA expression of colonic ZO-1 and Occludin. **D** Ttransepithelial resistance and FD4 permeability in the colon. **E** The number of goblet cells in the colon. (N = 6 mice per group, *P < 0.05, **P < 0.01 vs. the EA group)

From Fig. 9, we found that AM251 affected the effects of EA in repairing the intestinal barrier. IF showed a lower distribution of ZO-1 and Occludin in the EA + AM251 group (Fig. 9A). Moreover, the expression of ZO-1 at the protein level was decreased with AM251 exposure (P = 0.015 in the LEA + AM251 group and P = 0.002 in the HEA + AM251 group). The protein level of Occludin in the HEA + AM251 group was decreased significantly (P = 0.037) and the LEA + AM251 group showed a decreasing trend (Fig. 9B). Moreover, the mRNA levels of ZO-1 and Occludin were reduced (P = 0.005 and P = 0.026 in the LEA + AM251 group; P = 0.001 and P = 0.047 in the HEA + AM251 group, respectively) after the administration of AM251 (Fig. 9C). In addition, the TER of the EA + AM251 group was lower (P = 0.02 in the LEA + AM251 group and P = 0.006 in the HEA + AM251 group), and the FD4 permeability was increased in the EA + AM251 group (P = 0.015 in the LEA + AM251 group and P = 0.016 in the HEA + AM251 group; Fig. 9D). For goblet cells, the EA + AM251 group exhibited a significant decrease (P = 0.017 in the LEA + AM251 group and P = 0.002 in the HEA + AM251 group; Fig. 9E).

**Fig. 10:**
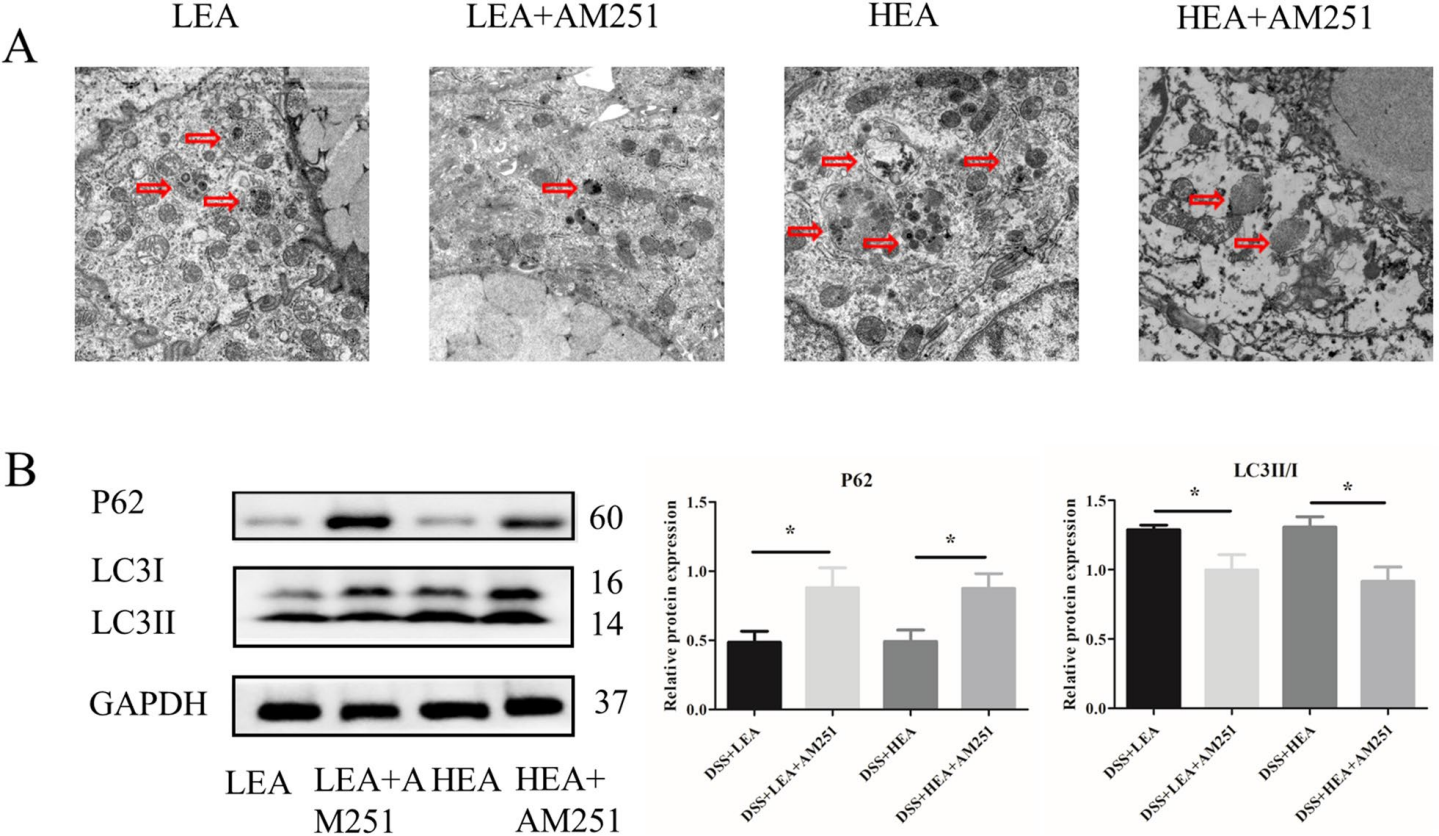
AM251 reversed the effects of EA on autophagy. **A** The autophagosome in the colon. **B** The protein levels of autophagy-related proteins in the colon (n = 6 mice per group, *P < 0.05 vs. the EA group)

As shown in Fig. 10, AM251 treatment also affected the degree of autophagy. The number of autophagosomes was reduced by AM251 (Fig. 10A). The expression of P62 was increased (P = 0.039 in the LEA + AM251 group and P = 0.018 in the HEA + AM251 group) and the level of LC3II/I was decreased (P = 0.046 in the LEA + AM251 group and P = 0.011 in the HEA + AM251 group, Fig. 10B) in the EA + AM251 group.

### FMT from EA could also improve colitis, repair the intestinal barrier, upregulate CB1, and enhance the degree of autophagy

**Fig. 11:**
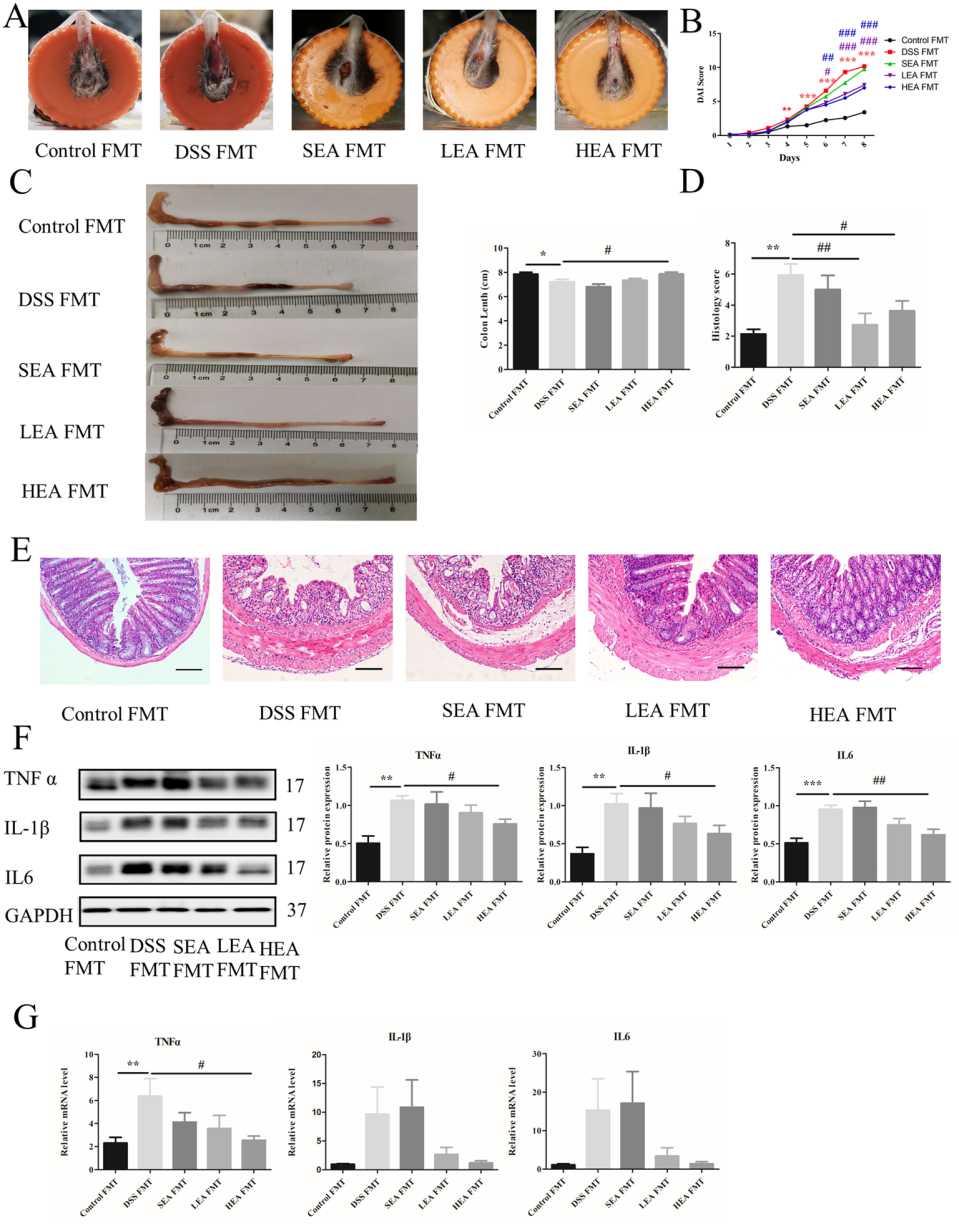
EA FMT alleviated colitis. **A** A representation of rectal bleeding in mice at Day 8. **B** Disease activity index scores from Day 1 to Day 8. **C** Colon length of mice. **D** Histological scores. **E** Representative HE staining images. Scale bar = 100 μm. **F** Colonic protein levels of TNFα, IL1β and IL6. **G** Relative mRNA expression of colonic TNFα, IL1β and IL6. Data are expressed as the mean ± SEM (n = 12 mice per group in **B**, **C**, n = 6 mice per group in **D**, **F**, **G** *P < 0.05, **P < 0.01, ***P < 0.001 vs. the control FMT group; ^#^P < 0.05, ^##^P < 0.01, ^###^P < 0.001 vs. the DSS FMT group)

The rectal bleeding in the DSS FMT group was more serious than that in the control FMT group and was relieved when EA FMT was administered (Fig. 11A). Moreover, the DAI scores of DSS FMT were increased at Day 4, and decreased with EA FMT at Day 6 (Fig. 11B). The length of the colon was shorter in the mice of the DSS FMT group (P = 0.018), and HEA FMT lengthened the colon (P = 0.016), while LEA FMT resulted in an increasing trend (Fig. 11C). Moreover, histological analysis showed that DSS FMT caused more serious histological injury such as severe mucosal damage and transmural leukocyte infiltration, and led to the increased histological scores (P = 0.001), and EA FMT resulted in partial recovery (P = 0.003 in the LEA FMT group and P = 0.028 in the HEA FMT group; Fig. 11D, E). For the expression of inflammatory factors, the protein levels of TNFα, IL-1β, and IL6 in the HEA FMT group were lower than those of the DSS FMT group (P = 0.042, P = 0.041, and P = 0.003, respectively), and the LEA FMT group only showed a decreasing trend (Fig. 11F). The mRNA results revealed that the expression of IL1β and IL6 in the EA FMT group exhibited a downwards trend compared with that in the DSS FMT group, and only the level of TNFα in the HEA FMT group (P = 0.01) was lower than that in the DSS FMT group (Fig. 11G).

**Fig. 12:**
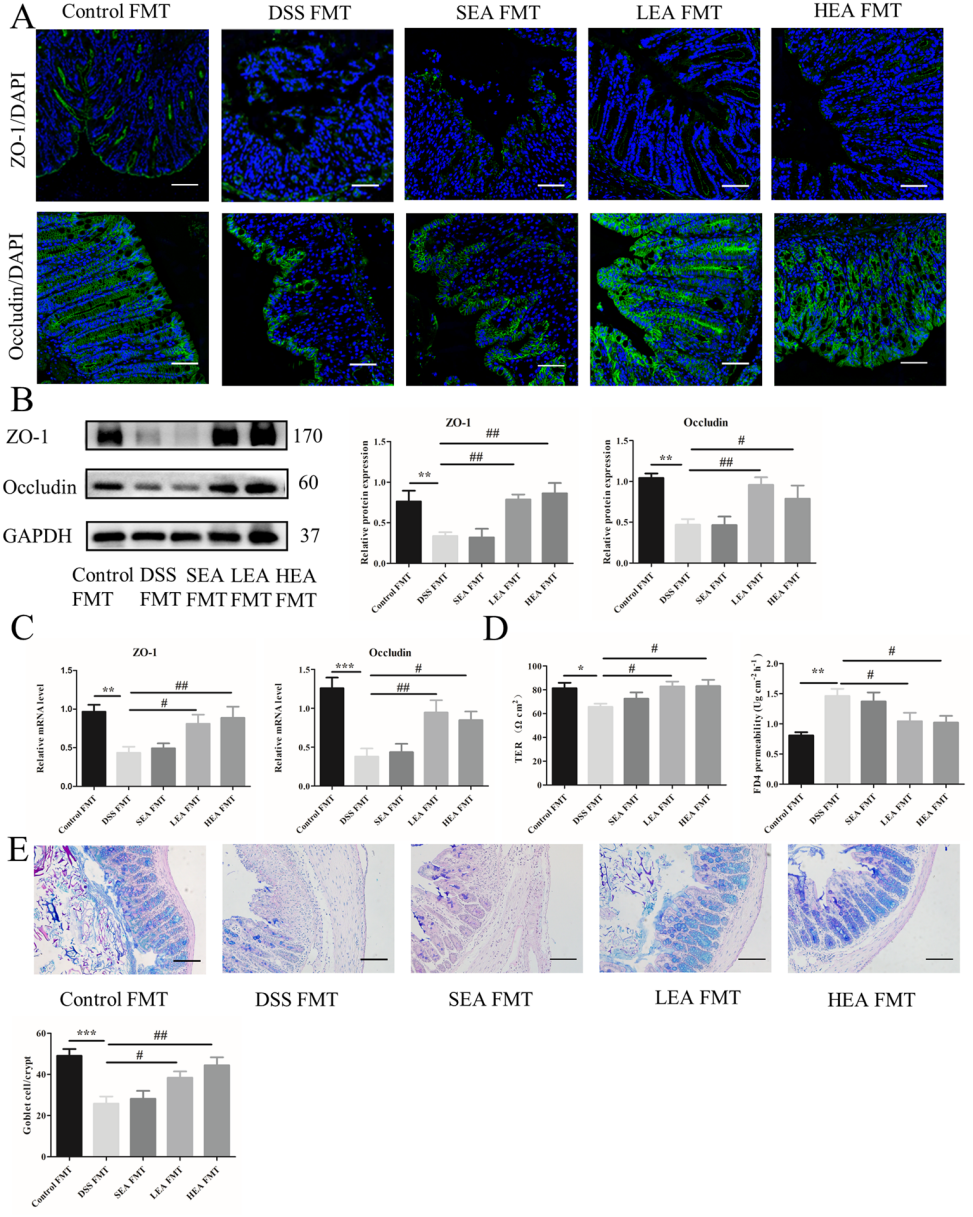
EA FMT improved the integrity of the intestinal barrier. **A** The distribution of ZO-1 and Occludin in colonic tissues. Scale bar = 100 μm. **B** The protein levels of colonic ZO-1 and Occludin. **C** The mRNA expression of colonic ZO-1 and Occludin. **D** Transepithelial resistance and FD4 permeability in the colon. **E** The number of goblet cells in the colon. (N = 6 mice per group, *P < 0.05, **P < 0.01, ***P < 0.001 vs. the control FMT group; ^#^P < 0.05, ^##^P < 0.01 vs. the DSS FMT group)

IF showed that the distribution of ZO-1 and Occludin in the DSS FMT group was decreased, and the EA FMT could partly recover this distribution (Fig. 12A). Western blot analysis showed that the expression of ZO-1 and Occludin in the EA FMT group was enhanced (P = 0.004 and P = 0.003 in the LEA FMT group; P = 0.001 and P = 0.039 in the HEA FMT group, respectively) compared with that in the DSS FMT group (Fig. 12B). We found similar results at the mRNA level (P = 0.016 and P = 0.004 in the LEA FMT group; P = 0.005 and P = 0.014 in the HEA FMT group, respectively; Fig. 12C). In addition, the TER of the DSS FMT group was lower (P = 0.022), and EA FMT increased this value (P = 0.013 in the LEA FMT group and P = 0.012 in the HEA FMT group). Moreover, EA FMT treatment decreased FD4 permeability (P = 0.021 in the LEA FMT group and P = 0.015 in the HEA FMT group) compared with that of the DSS FMT group (Fig. 12D). Similarly, the number of goblet cells was also increased in the LEA FMT (P = 0.018) and HEA FMT groups (P = 0.001; Fig. 12E).

**Fig. 13:**
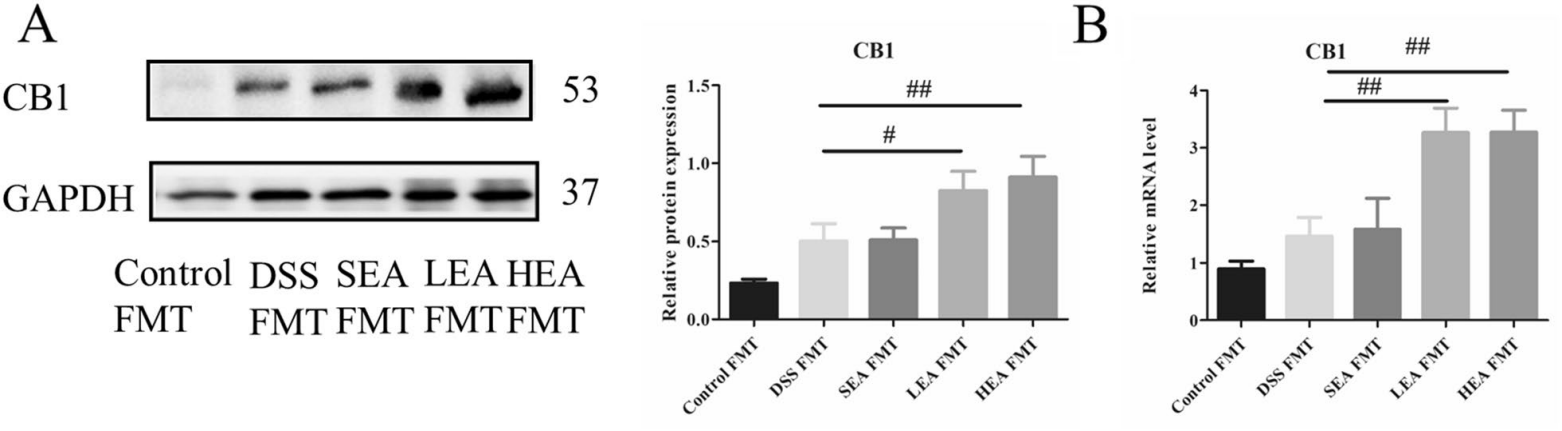
EA FMT upregulated the expression of CB1. **A** The protein level of colonic CB1. **B** The mRNA expression of colonic CB1. (n = 6 mice per group, ^#^P < 0.05, ^##^P < 0.01 vs. the DSS FMT group)

The expression of CB1 in the EA FMT group was also increased compared to that in the DSS FMT group at both the protein (P = 0.035 in the LEA FMT group and P = 0.009 in the HEA FMT group) and mRNA levels (P = 0.003 in the LEA FMT group and P = 0.003 in the HEA FMT group; Fig. 13).

**Fig. 14:**
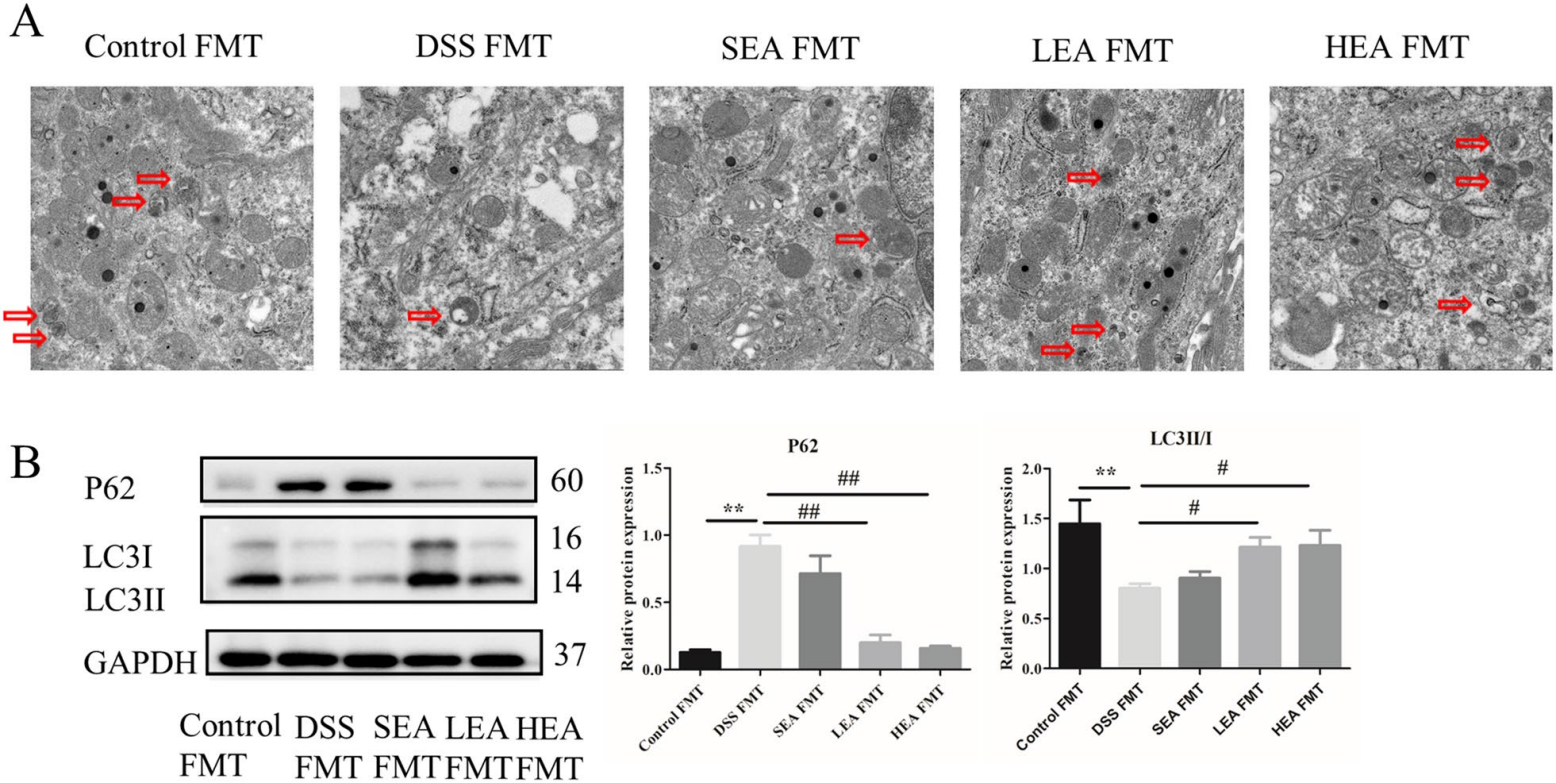
EA FMT strengthened the degree of autophagy. **A** The autophagosome in the colon. **B** The protein levels of autophagy-related proteins in the colon (n = 6 mice per group, **P < 0.01 vs. the control FMT group; ^#^P < 0.05, ^##^P < 0.01 vs. the DSS FMT group)

As shown in Fig. 14, we found a lower degree of autophagy in the DSS FMT group, such as a reduced number of autophagosomes (Fig. 14A), increased level of P62 (P = 0.001) and decreased level of LC3II/I (P = 0.003, Fig. 14B). The transplantation of feces from the EA group increased the number of autophagosomes, reduced the expression of P62 (P = 0.001 in the LEA FMT group and P = 0.002 in the HEA FMT group), and increased the level of LC3II/I (P = 0.045 in the LEA FMT group and P = 0.038 in the HEA FMT group).

## Discussion

In this study, our data showed that EA at the ST36 acupoint could repair the intestinal barrier, and further study demonstrated that EA reshaped the microbiota structure, upregulated CB1 expression and enhanced autophagy in DSS-induced acute colitis. Moreover, antagonizing CB1 could reverse the effects of EA, and FMT from the EA group could exhibit similar effects to EA. From the above results, we concluded that EA may repair the gut barrier to ameliorate intestinal inflammation via following mechanisms: modulating CB1 to enhance the level of autophagy through gut microbiota.

The gut barrier plays an important role in intestinal health. Dysfunction of the intestinal barrier is an important pathologic mechanism of colitis, which permits bacterial products and other antigens to cross the epithelium, leading to intestinal inflammation [21, 22]. Several studies have shown that the loss of tight junction proteins and increase in permeability facilitate the development of inflammatory infiltrates in colitis [23, 24]. Therefore, the gut barrier is regarded as an important target for the treatment of intestinal inflammation. As a part of complementary and alternative medicine, EA has been proven to be effective in the protection of the intestinal barrier in recent years. Our previous study showed that EA increased the levels of tight junction proteins, decreased intestinal permeability and bacterial invasion, and further relieved chronic colitis [5]. In acute colitis, Liu et al. showed that EA could preserve the gut barrier by upregulating the expression of tight junction proteins [4]. In the present study, we showed that EA repaired the gut barrier by increasing tight junction proteins and decreasing intestinal permeability, and further alleviated colonic inflammation.

The underlying mechanisms by which EA repaired the intestinal barrier were also investigated. Accumulating evidence has demonstrated that the endocannabinoid system is involved in intestinal homeostasis [25]. As a main member of the endocannabinoid system, CB1 is closely associated with repairing the intestinal barrier. It was reported that the CB1 agonist induced wound healing in colonic epithelial cell lines, indicating that CB1 activation improved the damaged intestinal barrier [26]. Similarly, cannabinoids, binding to CB1, inhibited the cytokine-induced increase in paracellular permeability in vitro and upregulated tight junction proteins [27]. In an in vivo study, CB1^−/−^ mice presented greater colonic barrier dysfunction characterized by greater paracellular permeability and bacterial translocation [28]. Therefore, CB1 is considered to be a promising target for the intestinal barrier. Several studies have found that EA exerts its therapeutic effect by upregulating the expression of CB1 in some noncolitis animal model [13, 29]. In our study, we first found that EA treatment could increase the expression of CB1 in acute colitis. Moreover, a CB1 antagonist was applied to further explore whether EA protects the gut barrier through the regulation of CB1. We found that the mice administered a CB1 antagonist showed a worse gut barrier and more serious colitis, which suggested that EA could repair the intestinal barrier by regulating the level of CB1.

We also studied the potential molecular mechanisms involved in CB1. Autophagy was reported to play an important role in maintaining the intestinal barrier. Induction of autophagy reduced paracellular permeability of the intestinal epithelium [30]. Correspondingly, inhibition of autophagy caused tight junction protein dysfunction and increased barrier permeability [31]. Notably, studies have noted that CB1 participates in the regulation of autophagy. Cannabidiol-induced autophagy was significantly inhibited by the administration of a CB1 antagonist [32]. Likewise, the selective CB1 agonist enhanced autophagic responses at both in vitro and in vivo experiments [33]. In this study, we found that the degree of autophagy was significantly decreased when the CB1 antagonist was administered, which suggested that CB1 may enhance autophagy and further repair the intestinal barrier. Notably, there has been no study on the relationship between EA and autophagy in colitis, and we found the benefits of EA in repairing hampered autophagy in DSS-induced acute colitis for the first time. In short, we showed that EA increased the level of CB1 to enhance the degree of autophagy, thereby protecting gut barrier integrity, and further alleviating colitis.

The potential mechanisms of EA in regulating the expression of CB1 were also explored. Several studies have shown that the gut microbiota can modulate the levels of CB1. Antibiotic-treated mice showed downregulation of CB1 [34], while probiotic supplement upregulated CB1 [15]. In our study, to further evaluate the role of the gut microbiota, we performed FMT experiments. The mice that received microbiota from the EA group also showed an increased level of CB1, which suggests that the gut microbiota largely mediates the regulatory effect of EA on CB1. In addition, Spearman correlation analysis showed that the norank_f_*Desulfovibrionaceae*, norank_f_norank_o_*Rhodospirillales*, and *Rikenellaceae*_RC9_gut_group, enriched in the EA group, were positively correlated with the expression of CB1, which indicates that the high enrichment of certain bacteria in the EA group may be connected with a high expression of CB1. Collectively, our results reveal that EA may increase the expression of CB1 by regulating the intestinal flora.

Currently, a few studies have shown that EA can change the composition of intestinal microbiota in acute colitis [4, 35, 36]. Similarly, in our study, EA could increase the flora diversity and restore the microbial community structure. What’s more, EA decreased the relative abundance of *Peptostreptococcaceae* and increased the relative abundance of *Lachnospiraceae*. At the genera level, the relative abundances of *Romboutsia* and *Turicibacter* were decreased with the treatment of EA. These results are basically consistent with the previous studies. In addition, KEGG analysis was performed to explore the effects of EA on the function of gut microbiota in this study, and we found that the EA group were significantly enriched with those microbial communities expressing functional genes associated with some metabolic pathways, which could be part of our ongoing research. Notably, compared with the previous studies, not only did we find changes in the microbiota with EA, we also performed FMT experiments to further verify that the altered microbiota can also exert the role of EA.

However, the mechanisms by which the gut microbiota affects the levels of CB1 are still unclear. In the EA group, the relative abundance of *Lachnospiraceae* was significantly increased, and the *Rikenellaceae*_RC9_gut_group, positively correlated with the CB1 level, was obviously enriched. *Lachnospiraceae* and *Rikenellaceae*_RC9_gut_group play an important role in the production of butyrate [37, 38]. Okumura et al*.* showed that butyrate could improve the intestinal hyperpermeability in septic rats, but CB1 antagonist weakened the effects of butyrate significantly [39], which indicated that butyrate could repair the intestinal barrier through CB1 signaling. In addition, some microbial metabolites have similar chemical structures to endocannabinoids, which can be identified as CB1 agonistic ligands and may promote the expression of CB1 [40, 41]. Collectively, we speculated that the increased or enriched abundance of certain bacteria after EA leaded to an upregulation of butyrate or other metabolites with cannabinoid-like structure, and further result in the promotion of CB1 level.

## Conclusions

In conclusion, the data of our study demonstrated that EA at ST36 could repair the intestinal barrier in DSS-induced acute colitis and identified the possible mechanisms: upregulating CB1 to enhance the autophagy degree via gut microbiota. These findings suggest that EA may be a promising therapeutic approach for the treatment of colitis.

## Supplementary Information

**Additional file 1: Figure S1.** AM251 downregulated the expression of CB1. **A** The protein level of colonic CB1. **B** The mRNA expression of colonic CB1. (n = 6 mice per group, *P < 0.05, **P < 0.01, ***P < 0.001 vs. the EA group).

## Data Availability

The 16S rRNA sequencing data have been deposited to the NCBI Sequence Read Archive and are available at the accession number PRJNA872832. The datasets supporting the conclusions of this article are included within the article.

## Abbreviations

EA: Electroacupuncture
DSS: Dextran sulfate sodium
CB1: Cannabinoid receptor 1
FMT: Fecal microbiota transplantation
DAI: Disease activity index
TER: Transepithelial resistance
IF: Immunofluorescence
LDA: Linear discriminant analysis
