# Supplementary figures and images for: Electroacupuncture repairs intestinal barrier by upregulating CB1 through gut microbiota in DSS-induced acute colitis
Source: Chin Med. 2023 Mar 10;18:24. doi: 10.1186/s13020-023-00733-9 (PMC9999655; doi:10.1186/s13020-023-00733-9)

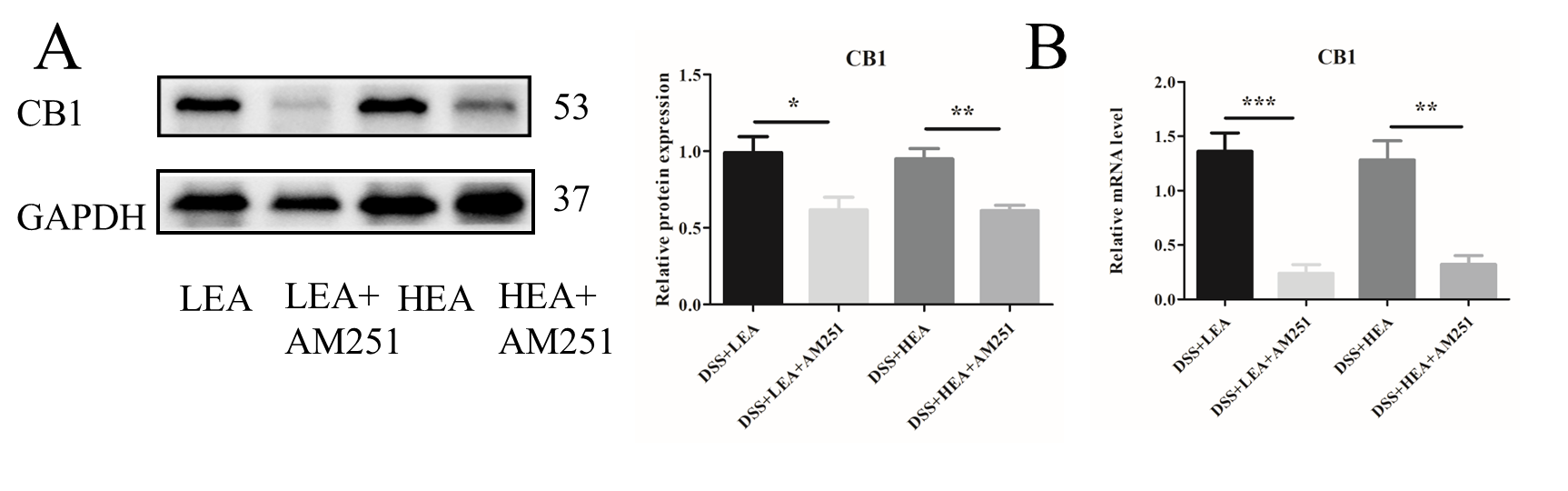

Supplement: Supplementary file 1 — Additional file 1: Figure S1. AM251 downregulated the expression of CB1. A The protein level of colonic CB1. B The mRNA expression of colonic CB1. (n = 6 mice per group, *P < 0.05, **P < 0.01, ***P < 0.001 vs. the EA group). [file 13020_2023_733_MOESM1_ESM.tif]
